# Editorial: Exploring combination therapies for effective obesity and metabolic syndrome management

**DOI:** 10.3389/fendo.2026.1824139

**Published:** 2026-04-17

**Authors:** Puspha Sinnayah, Michael Mathai

**Affiliations:** 1 First Year College, Victoria University, Melbourne, VIC, Australia; 2 Institute for Health and Sport, Victoria University, Melbourne, VIC, Australia

**Keywords:** bariatric surgery, combination (combined) therapy, GLP - 1, metabolic syndrome & type II diabetes, obesity

Obesity remains one of the most complex and rapidly increasing global health challenges, contributing profoundly to non-communicable diseases such as type 2 diabetes (T2D), cardiovascular disease (CVD), and metabolic syndrome. Since 1975, the worldwide prevalence of obesity has tripled, and projections suggest nearly one billion adults—close to one-fifth of the global population—will be affected by 2025 if current trends continue. This alarming trajectory highlights the inadequacy of existing strategies and the urgent need for multi-dimensional therapeutic interventions. While pharmacological monotherapies, such as GLP-1 receptor agonists, have delivered notable progress, their limited long-term efficacy and tolerability underscore the necessity of exploring combination therapies that simultaneously modulate multiple metabolic pathways.

Recent research advances illustrate a clear paradigm shift—from isolated pharmacological targets toward integrated treatment approaches that align with the multi-systemic nature of obesity. Park et al. emphasize in a review article, the evolving pipeline of anti-obesity pharmacotherapies targeting multiple hormonal pathways, including GLP-1, GIP, and glucagon receptors. This polyagonist approach reflects a more physiologically coherent strategy, designed to reproduce the natural hormonal complexity regulating appetite, energy consumption, and glucose metabolism. Such research supports the broader rationale for developing combination therapies that achieve enhanced weight loss by influencing complementary central and peripheral mechanisms, thus optimizing efficacy while mitigating side effects.

The emergence of novel oral GLP-1 receptor agonists such as danuglipron and orforglipron, as documented by Zhou et al. in a systematic review, reinforces this evolution. Their findings suggest that orally administered agents can match the weight loss benefits of injectable formulations, expanding accessibility and adherence options for patients. When integrated into combination regimens, these compounds may anchor a new generation of non-invasive, multitarget therapies, particularly suited for scalable clinical use. However, the long-term safety, durability, and optimal combination with adjunct drugs or behavioral strategies remain areas demanding systematic exploration.

Beyond pharmacology, the synergistic potential of combining biological and lifestyle interventions is increasingly documented. Shang et al. demonstrated that natural compounds such as Ramulus Mori (Sangzhi) (SZ-A) alkaloids ameliorate obesity by modulating gut microbiota composition and bile acid metabolism, in a high-fat diet rat model. SZ-A significantly reduced body weight, improved lipid profiles, and alleviated liver injury in high-fat diet–induced obese rats, demonstrating clear metabolic benefits. It reshaped gut microbiota composition and regulated bile acid metabolism, decreasing harmful bile acids while activating FXR-FGF15 and TGR5-GLP-1 pathways linked to metabolic control. This microbiota-mediated axis presents an additional layer of therapeutic targeting, suggesting that future combination therapies could integrate microbiome modulation to enhance metabolic resilience. Similarly, de Waal et al. highlighted in a review, that combining bariatric or endoscopic interventions with pharmacotherapies or probiotics enhances weight loss durability and improves metabolic outcomes. These findings illustrate that integrated approaches—linking surgical, pharmacological, and microbial modulation—could deliver longer-lasting metabolic benefits than any single modality.

Lifestyle synergy remains pivotal in amplifying pharmacological results. Paternò et al. observed in a clinical trial, that adherence to the Mediterranean diet potentiates the effects of tirzepatide, a dual GLP-1/GIP agonist, improving not just weight loss but also insulin sensitivity and adiposity indices. Higher diet adherence was independently associated with lower insulin resistance and greater improvements in visceral adiposity. This underscores that obesity management must transcend mere calorie restriction or pharmacotherapy, positioning combination strategies as multidimensional interventions encompassing neuroendocrine, metabolic, and behavioral facets. Taken together, such evidence aligns with a holistic model of obesity care—one that acknowledges the dynamic interplay between biological systems and environmental influences.

From a wider metabolic perspective, insights from Huang et al. in a systematic review, on the combination of fenofibrate and statins in patients with diabetes and hyperlipidemia serve as a relevant analogy. Dual-target therapy has been shown to work better than single-drug treatment, suggesting that targeting multiple pathways at once can improve blood sugar and lipid control. Yet, the caution against increased muscular and hepatic adverse events highlights the essential balance between therapeutic gain and safety. This principle must guide future development and clinical application of anti-obesity combination regimens.

Across these contributions, several unifying directions emerge that reflect the evolving landscape of obesity research and treatment. First, there is growing recognition of mechanistic diversity, with future therapeutic strategies likely to integrate multiple physiological pathways—including neurohormonal regulation, gut–brain signaling, microbiota modulation, and hepatic lipid metabolism—to address the complex and interconnected drivers of obesity. At the same time, advances in translational precision are reshaping therapeutic development; tools such as systems biology, receptor mapping, and computational modeling now enable more rational design of combination therapies, moving beyond empirical drug pairing toward mechanistically informed interventions. These developments also support a shift toward personalized treatment, where patient-specific characteristics—such as metabolic phenotype, comorbidities, genetic predisposition, and microbiome composition—may guide tailored therapeutic regimens that optimize efficacy and minimize adverse effects. Finally, the studies highlight the importance of a continuum-of-care approach, emphasizing that sustainable obesity management requires coordinated integration of pharmacological treatments with behavioral strategies and, where appropriate, procedural interventions. Together, these directions signal a broader transition toward integrative, mechanism-driven, and patient-centered strategies that more effectively reflect the multifactorial nature of obesity. Such approaches reflect a maturation of the field—from viewing obesity as a monolithic disorder to recognizing it as a heterogeneous, systems-level disease demanding modular solutions.

Situating these goals within a broader scientific and public health framework, this Research Topic reflects a shift toward precision and systems-based medicine in obesity management. Rather than viewing obesity as a static accumulation of excess adiposity, the emerging paradigm conceptualizes it as a dynamic disorder involving dysregulated hormonal feedback, altered gut-brain signaling, and chronic metabolic inflammation. By leveraging combination therapies that act along these multiple axes, researchers can design interventions that are not only more effective but also personalized—addressing the specific biological and behavioral determinants that underlie obesity in individual patients.

The global implications are profound. As healthcare systems grapple with increasing obesity prevalence and its downstream complications, combination therapy research offers a pathway toward equitable, sustainable, and scalable solutions. Effective combination regimens—whether integrating advanced dual receptor agonists, microbiota modulators, or supportive dietary frameworks—could reduce reliance on high-risk surgical interventions and provide accessible pharmacological alternatives across socioeconomic contexts. Moreover, these therapies could reshape obesity management from reactive weight-loss interventions to proactive metabolic health restoration. In conclusion, the exploration of combination therapies represents a critical and timely evolution in obesity research. By bridging molecular pharmacology, nutritional science, microbiome biology, and clinical practice, this approach embodies a truly integrative response to a multifaceted disease. The convergence of evidence from diverse studies—from receptor polyagonists and gut microbiota modulation to lifestyle integration and surgical adjuncts—signals a transformative moment in the field.

Future directions also include the development of multimodal treatment models that combine pharmacological therapies with procedural interventions when appropriate. Integrating anti-obesity medications and microbiome-modulating strategies with bariatric or endoscopic procedures may improve long-term weight maintenance and reduce metabolic relapse following surgery. At a broader translational level, advances in systems biology and computational modeling will likely guide more rational design of combination therapies, allowing researchers to identify synergistic targets across endocrine, neural, and metabolic networks. Ultimately, these innovations support the movement toward personalized obesity medicine, where treatment regimens are tailored according to patient phenotype, metabolic comorbidities, genetic risk, and even microbiome profiles. By aligning pharmacological, behavioral, microbiological, and procedural strategies within a coordinated continuum of care, future research aims to deliver more sustainable, effective, and equitable solutions for obesity and its associated cardiometabolic complications.

